# Vineyard water status assessment using on-the-go thermal imaging and machine learning

**DOI:** 10.1371/journal.pone.0192037

**Published:** 2018-02-01

**Authors:** Salvador Gutiérrez, María P. Diago, Juan Fernández-Novales, Javier Tardaguila

**Affiliations:** Instituto de Ciencias de la Vid y del Vino (University of La Rioja, CSIC, Gobierno de La Rioja), Finca La Grajera, Ctra. Burgos Km. 6, 26007 Logroño, Spain; Universidade do Minho, PORTUGAL

## Abstract

The high impact of irrigation in crop quality and yield in grapevine makes the development of plant water status monitoring systems an essential issue in the context of sustainable viticulture. This study presents an on-the-go approach for the estimation of vineyard water status using thermal imaging and machine learning. The experiments were conducted during seven different weeks from July to September in season 2016. A thermal camera was embedded on an all-terrain vehicle moving at 5 km/h to take on-the-go thermal images of the vineyard canopy at 1.2 m of distance and 1.0 m from the ground. The two sides of the canopy were measured for the development of side-specific and global models. Stem water potential was acquired and used as reference method. Additionally, reference temperatures T_dry_ and T_wet_ were determined for the calculation of two thermal indices: the crop water stress index (CWSI) and the Jones index (I_g_). Prediction models were built with and without considering the reference temperatures as input of the training algorithms. When using the reference temperatures, the best models casted determination coefficients R^2^ of 0.61 and 0.58 for cross validation and prediction (RMSE values of 0.190 MPa and 0.204 MPa), respectively. Nevertheless, when the reference temperatures were not considered in the training of the models, their performance statistics responded in the same way, returning R^2^ values up to 0.62 and 0.65 for cross validation and prediction (RMSE values of 0.190 MPa and 0.184 MPa), respectively. The outcomes provided by the machine learning algorithms support the use of thermal imaging for fast, reliable estimation of a vineyard water status, even suppressing the necessity of supervised acquisition of reference temperatures. The new developed on-the-go method can be very useful in the grape and wine industry for assessing and mapping vineyard water status.

## Introduction

Water utilization has become a critical issue in sustainable agriculture, and because of reasons such as water scarcity and climate change, its management is increasingly required to be more accurate. In viticulture, irrigation has a direct impact on vine yield and grape quality, so the implementation of precision watering systems could be considered as useful tools for the precise application of water regimes based on water status reports of the vineyard plot [1]. Several authors have studied different physiological parameters for the monitoring of the plant water status [2–4], and the direct measurement of the plant has been widely put into action for the measurement of the water status, instead of moisture measurements of the soil [5]. A key factor in the precise water management in vineyards may lie on the development of reliable tools for assessing the vine water status and its spatial variation directly in the field. This goal is virtually unreachable by the classical measurement devices due to their labor demanding, time-consuming nature and necessity of expert and trained personnel.

Infrared thermography is a technique based on the relationship between leaf stomatal closure or aperture and its surface temperature [6]. When leaf transpires, water is lost through stomata and leaf temperature decreases. However, if transpiration stops, leaf temperature increases as no heat dissipation is ocurring. Likewise, the temperature of the canopy has been correlated with specific plant physiological parameters, such as the stomatal conductance (g_s_) [5, 7–9]. Technological advances in thermal imaging have delivered new opportunities in the acquisition of plant thermal responses to water status variations [8, 10–12]. In this fashion, thermal cameras are very prone to be used as portable devices for the estimation of plant water status and to help in the set up of irrigation schedules [10, 13, 14]. Still, further advancement based on canopy temperature has led to the development of thermal indices, such as the crop water stress index (CWSI) [15] and the conductance index (I_g_) [6], that—by previously taking reference temperatures—seek for the diminishing of environmental variations that could affect the canopy temperature. As a result, several studies exploring semi-supervised irrigation scheduling system based on thermal information have been recently published [16–18]. Nevertheless, in the majority of the cases, the computation of thermal indices relies on mandatory acquisitions of edge temperatures, typically T_wet_ and T_dry_ for minimal and maximal temperatures, respectively, with the need of inducing extreme temperatures using adapted leaves or special devices [9]. This hinders the development of fully automated thermal methods for the estimation of plant water status. Hence, the overcome of the measurement of reference temperatures would stand for a substantial improvement in this matter in order to automate this technology in industrial applications.

In-field, non-invasive assessment of grapevine water status and its variability within the vineyard would be a valuable tool in precision viticulture. Correlation analyses between thermal indices and physiological parameters such as g_s_ and stem water potential Ψ_stem_ have been carried out in the field using non-destructive portable sensors in commercial vineyards bringing compelling correlation levels [19, 20]. The main advantages these methods brought were the easy implementation and processing and immediate response. Nonetheless, it is needed for the device to be constantly managed by active human resources and it is only possible to make measurements on individual specific spots, factors that would make it difficult to expand the measurements for the fast monitoring of a whole vineyard. This main pitfall has been partially resolved by aerial thermal imaging [21–24], that successfully covered large extensions of vineyard. However, in the majority of cases the aerial point of view comes with a reduced spatial resolution in the measurements that shrink several meters of the canopy into a few number of pixels, losing a definite amount of information. This brings the opportunity of developing thermal systems for the grapevine water status estimation capable of gathering detailed canopy information—from a close lateral point of view—and to cover large areas for the monitoring of a vineyard variability—using stop-and-go and on-the-go approaches, as already attempted by other works [25, 26]. Moreover, the removal of constant human supervision would allow the possibility of mounting automatic acquisition systems in on-work agricultural vehicles.

The demonstrated high level of performance machine learning has provided in a vast number of problems from very different nature presents it as one of the most important algorithm-provider research field. Regardless the area, if the problem can be properly modeled into an adequate input, machine learning algorithms could be very suitable for the discovering of underlying rules and latent connections between the provided information, for the categorization into specific identities (classification) or the estimation of real numbers (regression) [27, 28]. In recent years, several viticulture-related problems have been addressed using data mining and machine learning approaches [29–33]. Rotation forests [34] are prediction algorithms based on ensemble learning methods that can perform classification or regression tasks depending on the tree-based algorithm provided. Rotation forests build several trees after making use of principal component analysis to the randomly split input attributes.

The goal of this study was to develop a new on-the-go system involving thermal imaging and machine learning to assess the vineyard water status. Specifically, the objective was not only to analyze and find high levels of correlations between thermal information and physiological water status parameters, but to provide full trained, robust prediction models. These are fed with a huge amount of data from a wide period of time that covered an entire campaign, and are capable to return reliable estimations for the characterization of a whole vineyard plot and to be used in the irrigation decision making process.

## Materials and methods

### Experimental layout

**Fig 1:**
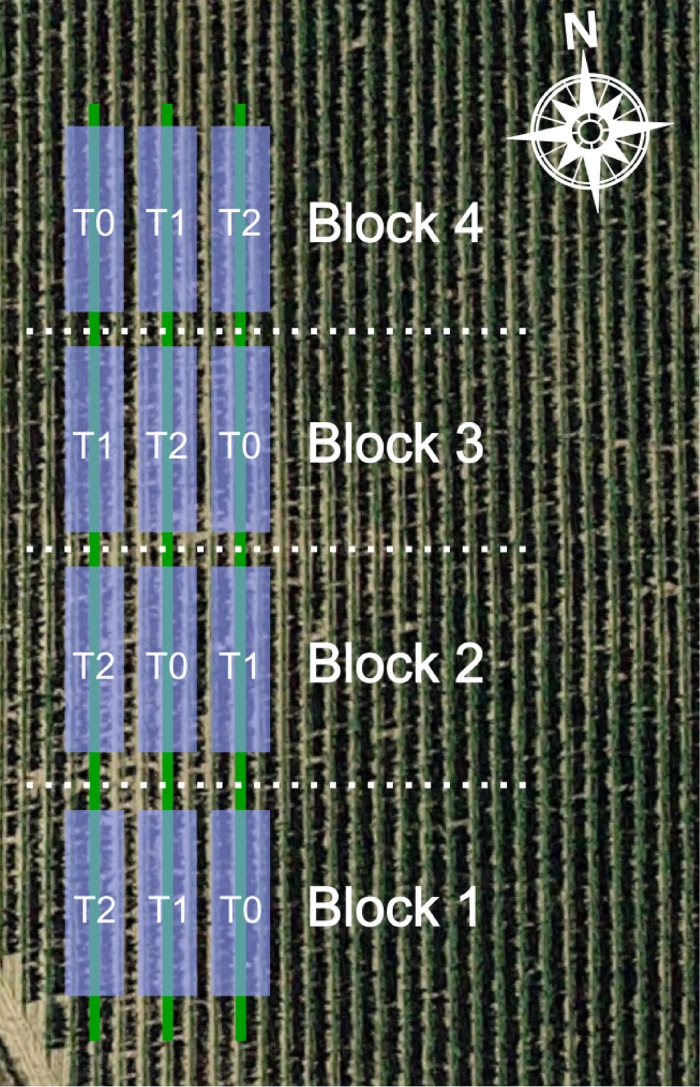
Experimental layout and water treatment replications. Each one of the four horizontal blocks comprised the three different water regimes applied. T0: full irrigation treatment. T1: moderate irrigation treatment. T2: no irrigation treatment.

The experiment was performed throughout seven weeks from early-July to early-September, 2016, in a 5 ha commercial vineyard of Tempranillo variety (*Vitis vinifera* L.) under permission of its owner. The vineyard plot was situated in Tudelilla, La Rioja, Spain (Lat. 42° 18’ 18.26”, Long. -2° 7’ 14.15”, Alt. 515 m). Grapevines, planted in 2002, were grafted on rootstock R-110 and trained to a vertically shoot-positioned (VSP) trellis system, having a North-South row orientation at 2.60 × 1.20 meters inter and intra row distances. To induce an extensive level of variability in the sample units and to obtain better trained prediction models, three different water regimes were deployed in a randomized complete block design [35] with four blocks (Fig 1). The three water treatments were:

- T0, full irrigation: two parallel water pipelines providing 6 L/h.
- T1, moderate irrigation: one water pipeline providing 3 L/h.
- T2, no irrigation: the plants were not irrigated during the whole experiment.

Irrigation was scheduled to be applied two hours per day, five days a week.

**Fig 2:**
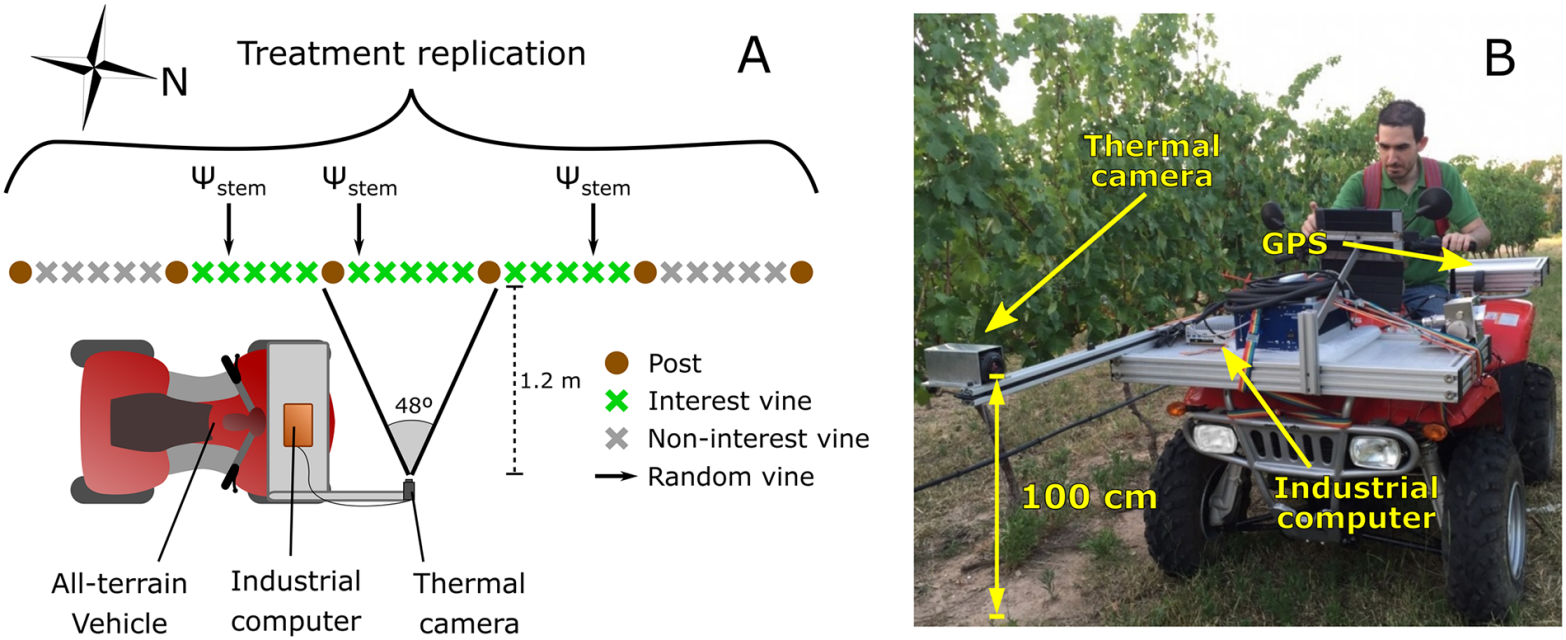
Detail from a treatment replication and thermal system (A) and picture of the actual on-the-go system (B). The thermal camera was connected to an industrial computer and controlled by the driver.

Each treatment involved four replications, therefore 12 different combinations of treatment and replication were present in the vineyard and located in three different parallel, equally-distanced vine rows. For each replication, comprising 25 plants (around 25 m of length), only the 15 middle ones were the ones in which the measurements were taken. The first and last groups of five plants were discarded to avoid edge effect (Fig 2A).

### Acquisition of thermal images

On-the-go thermal measurements were acquired using a thermal camera (FLIR A35, FLIR^®^ Systems, Inc., Bilerica, MA, USA) that was mounted in the front part of an all-terrain vehicle at a height from the floor of 1 m (Fig 2B) and connected to an industrial computer. The individual in this manuscript has given written informed consent (as outlined in PLOS consent form) to publish these case details. The camera was focused to the left at a distance from the canopy of approximately 1.2 m and with 48° × 39° horizontal and vertical field of views (FOV, Fig 2A), respectively. This distance and FOVs provided images covering canopy scenes of 1.07 m horizontally and 0.85 m vertically, approximately. Acquisition of the thermal images, at 60 frames per seconds (FPS), was performed in both east and west sides of the canopy at an average speed of 5 km/h at solar noon (between 14:00 and 15:00 hours, local time).

### Thermal images processing

At a recording framerate of 60 FPS, a treatment replication’s length of 25 m and an average speed of 5 km/h, the number of thermal frames captured per each replication was approximately 1080. In order to reduce the amount of redundant information, not every frame from the recordings was used in the processing of the images—only those frames having no overlapping were thus used for the calculation of different thermal statistics. If a treatment block of 25 m length is covered by 1080 frames, and each frame displayed approximately a horizontal length of 1.07 m, one out of 46 consecutive frames would have no overlapping. Thus, each treatment replication consisted of roughly 23 frames, and only the 14 middle ones were used since they comprised the 15 middle plants of the replication (the plants in which the water status measurements were taken).

To support the thermal images segmentation and develop different canopy temperature indices, T_wet_ and T_dry_ reference temperatures were acquired using an evaposensor (Skye Instruments, Llandrindod Wells, UK) having two artificial leaves: a dry one (dry reference) and another one covered with a black cotton wick and receiving continuous water absorption for the wet reference [36]. Reference temperatures were acquired once per each measurement day.

**Fig 3:**
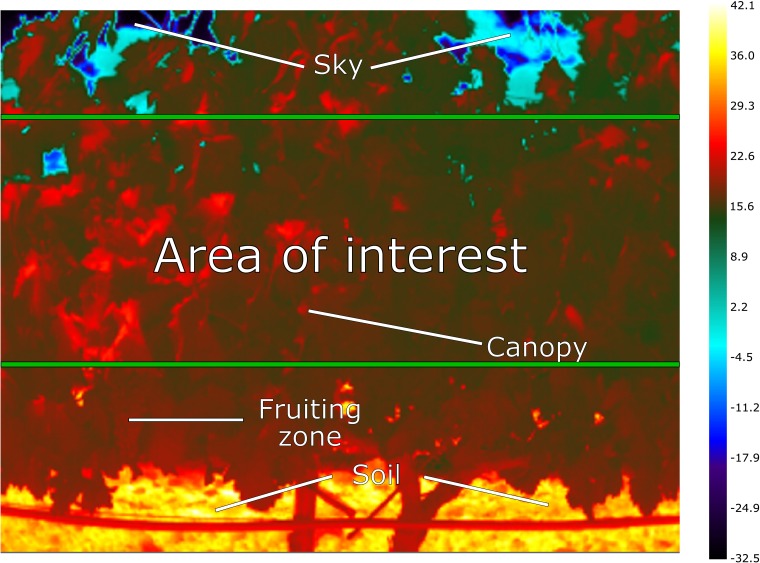
Canopy thermal image taken on-the-go at 5 km/h. The middle section between the green bars was defined as the area of interest. Total image resolution was 320 × 256 pixels, while the area of interest had a size of 320 × 135 pixels. The temperature values are expressed in degrees Celsius.

To remove the influence of the sky, soil and fruiting zone in the top and bottom part of the thermal images respectively, the middle section of the thermal images was selected, named as area of interest, and used for the calculation of the different statistics and thermal indices (Fig 3). The area of interest had a size of 320 × 135 pixels, from a total image resolution of 320 × 256 pixels.

The average temperature (T_canopy_), the median and the standard deviation were the statistics extracted from the thermal frames. Additionally, two thermal indices, the crop water stress index (CWSI) and the conductance index (I_g_) were calculated, using the reference temperatures, as follows, according to [37] and [6], respectively:

$$CWSI=\frac{T_{canopy}-T_{wet}}{T_{dry}-T_{wet}}$$ (1)

$$I_{g}=\frac{T_{dry}-T_{canopy}}{T_{canopy}-T_{wet}}$$ (2)

FLIR ResearchIR 4.40 (FLIR^®^ Systems, Inc., Billerica, MA, USA) and MATLAB 8.5.0 (The MathWorks Inc., Natick, MA, USA) were used for the processing of the thermal images.

### Measurement of grapevine water status

Plant water status measurements were also performed at solar noon (between 14:00 and 15:00 hours, local time) simultaneously to thermal image acquisition. During the whole campaign, the air temperature, relative humidity (for vapor pressure deficit (VPD) calculation [38]) and incoming solar irradiance were additionally recorded in the same hours from a weather station located in the vineyard. The reference method used for the measurement of the plant water status was the midday stem water potential Ψ_stem_. As shown in Fig 2A, a random vine was selected from each group of five plants (of the 15 middle ones in each replication) and its Ψ_stem_ was measured upon a leaf taken from the central part of the canopy. A Scholander pressure bomb (Model 600, PMS, Instruments Co., Albany, NY, USA) was used for the stem water potential determination [39]. The selected leaves were covered with aluminum foil bags to drive them into dark adaptation one hour before the measurements.

With 12 different replications and three selected plants per block, a total of 36 measurements of the plant water status were performed per day. Therefore, since the study was conducted across seven different weeks, and one day per week, a total of 252 water status measurements were used for the training of the prediction models.

### Machine learning algorithms and model development

Two different approaches were attempted for the extraction of the statistics that trained the prediction models: the use of T_dry_ and T_wet_ reference values for segmentation of the area of interest (the removal of all temperatures outside the T_wet_ and T_dry_ range) and calculation of thermal indices; and the extraction of the same statistics without segmentation and avoiding the calculation of the thermal indices.

A combination of rotation forests and decision trees (reduced error pruning tree, Weka’s REPTree implementation [40]) was used for the development of the regression models. Two kind of models were built:

- **Using T_dry_ and T_wet_ reference temperatures and thermal indices**, with the following input attributes: air temperature, average canopy temperature, median of the canopy temperature, standard deviation of the canopy temperature, CWSI and I_g_.
- **Avoiding the use of reference temperatures and thermal indices**, with the following input attributes: air temperature, average canopy temperature, median of the canopy temperature and standard deviation of the canopy temperature.

In both cases, the Ψ_stem_ was used as the plant water status reference value to predict.

Side-specific models for east and west sides of the canopy were individually built, each one including 252 samples. Additionally, a global dataset comprising samples from both east and west sides of the canopy was also generated. This global set was intended to be as similarly represented as those datasets from east and west side, therefore 126 samples from the east dataset and the same amount from the west dataset were picked to build a global set with 252 samples. This selection was pseudorandomly performed, taking the same amount of samples per canopy side, water regime and measurement day, in order to build a model from a well-represented input data.

In order to provide a complete set of performance statistics, calibration, 10-fold cross-validation and external prediction were carried out. The datasets were not completely used for the training of the regression models, but they were split in a 80-20 ratio, labeling the two originated subsets as train (80% of the original dataset) and test (the remaining 20%) sets. This division was not totally random. Samples representing the whole range of the stem water potential values obtained were extracted to the test set, also taking the same number of samples per measurement day. The calibration and cross validation were performed using the train set, while the prediction was achieved by training the model with the train set and predicting the test set.

## Results

### Vineyard status overview

A wide range of stem water potential values was covered throughout the whole experiment, obtaining Ψ_stem_ values at midday of the 252 samples from -2.05 MPa—meaning high level of water stress—to -0.40 MPa—no stress. The mean and standard deviation Ψ_stem_ values of -1.23 MPa and 0.298 MPa confirmed a high level of variability desirable for the development of representative plant water status prediction models.

Table 1 shows an overview by days in terms of VPD, incoming solar irradiance, canopy temperatures recorded by thermal imaging and Ψ_stem_ values.

**Table 1:** Mean values according to measurement days for vapor pressure deficit (VPD), incoming solar irradiance, canopy temperatures (by canopy side and irrigation treatment) and stem water potential (Ψ_stem_) determined at solar noon, between 14:00 and 15:00. *N* = 12 per irrigation treatment for the statistical tests of canopy temperatures and Ψ_stem_ (MPa).

		Jul, 6	Jul, 13	Jul, 20	Jul, 28	Aug, 11	Aug, 23	Sep, 8
**VPD (kPa)**		2.71	1.11	2.49	3.22	1.77	3.43	1.28
**Irradiance (W/m^2^)**		890.95	631.82	348.61	895.11	841.89	827.01	291.30
**East temp. (°C)**	**T0**	20.7	15.6	24.6	23.5 A	17.2	25.4 A	19.4 A
	**T1**	22.2	16.3	25.1	24.9 B	16.8	27.1 B	19.6 A
	**T2**	22.1	16.4	24.8	23.2 A	16.7	27.9 B	20.5 B
		*n.s.*	*n.s.*	*n.s.*	**	*n.s.*	***	***
**West temp. (°C)**	**T0**	22.5	16.0	24.7	25.2	16.8 A	26.8 A	20.4 A
	**T1**	23.8	16.9	25.2	27.0	17.4 B	28.5 B	20.5 A
	**T2**	22.6	16.1	25.4	25.5	18.0 C	29.1 B	21.1 B
		*n.s.*	*n.s.*	*n.s.*	*n.s.*	***	***	**
**Ψ_stem_ (MPa)**	**T0**	-0.97 A	-1.04 A	-1.00 A	-1.18 A	-1.19 A	-1.13 A	-0.56 A
	**T1**	-1.15 B	-1.28 B	-1.25 B	-1.26 AB	-1.47 B	-1.41 B	-0.96 B
	**T2**	-1.14 B	-1.22 B	-1.31 B	-1.31 B	-1.52 B	-1.65 C	-1.76 C
		***	***	***	**	***	***	***

Jul: July. Aug: August. Sep: September.

Dissimilar capital case letters within columns represent statistically different means among east and west temperatures and Ψ_stem_, using Tukey’s range test at a significance level p = 0.05.

*n.s.*: not significant (p ≥ 0.05);

**: *p* < 0.01;

***: *p* < 0.001.

VPD values ranged from 1.11 kPa (July 13th) to 3.43 kPa (August 23rd) while irradiance values were found to be above 800 W/m^2^ for most of the measuring dates, with the exception of the 13th and 20th of July, and the 8th of September. The values observed in the last date can be explained by the generalized meteorological behavior in the end of the season, as a decrease of irradiance and temperatures is prone to be found. Canopy temperatures were generally distinguishable between treatments, presenting slightly higher values in the west side. Additionally, the water status values presented an increasing trend throughout the summer as a consequence of season progress and water treatments’ effectiveness.

Statistically significant differences between the temperatures of the irrigation treatments were not found for either east or west sides in the first three days, while they were present in the last two ones. In July 28th, the east side of the canopy casted significant differences, while the west side did not, but the opposite occurred in August 11th. In the case of Ψ_stem_, all the dates presented statistically significant differences between the most extreme irrigation treatments (T0 and T2) while for the last two dates all three treatments were significantly different.

### Vineyard water status prediction models using T_dry_ and T_wet_ reference temperatures

The performance statistics of the prediction models trained with thermal indices are summarized in Table 2 for east and west models, and additionally for the global model trained with samples from both sides of the canopy.

**Table 2:** Performance statistics for the stem water potential (Ψ_stem_) prediction models using T_dry_ and T_wet_ reference temperatures.

	N = 200				N = 50	
	Calibration		Cross validation		Prediction	
Canopy side	R^2^	RMSE	R^2^	RMSE	R^2^	RMSE
**East**	0.83	0.139	0.61	0.190	0.57	0.206
**West**	0.81	0.148	0.57	0.202	0.58	0.204
**Global**	0.73	0.182	0.39	0.237	0.52	0.233

A 10-fold cross validation was used.

N: number of samples. RMSE: root mean square error (MPa). East: dataset having all the samples from the east side of the canopy. West: dataset having all the samples from the west side of the canopy. Global: dataset having non-repeated samples from both east and west sides of the canopy.

For the models built with the thermal information from the east side of the canopy, determination coefficients R^2^ of 0.83 and 0.61 were obtained for calibration and cross validation, casting RMSE values of 0.139 MPa and 0.190 MPa, respectively (Table 2). The performance of the prediction stage did not lied far from that of the cross validation, producing a R^2^ value of 0.57 and a RMSE of 0.206 MPa.

The results given by the west side models remained fairly similar, with a calibration R^2^ of 0.81 (RMSE of 0.148 MPa) and a cross validation R^2^ of 0.57 (RMSE of 0.202 MPa), being the latter slightly lower than that from the east side models. The prediction result was 0.58 for the determination coefficient with a root mean square error of 0.204 MPa (Table 2).

The performance achieved by the global models was lower in all the stages: calibration, cross validation and prediction, although in this last case the determination coefficient was closer to the ones from east and west models, as opposed to calibration and cross validation. Nevertheless, the RMSE values of cross validation and prediction remained very similar: 0.237 and 0.233 MPa respectively.

**Fig 4:**
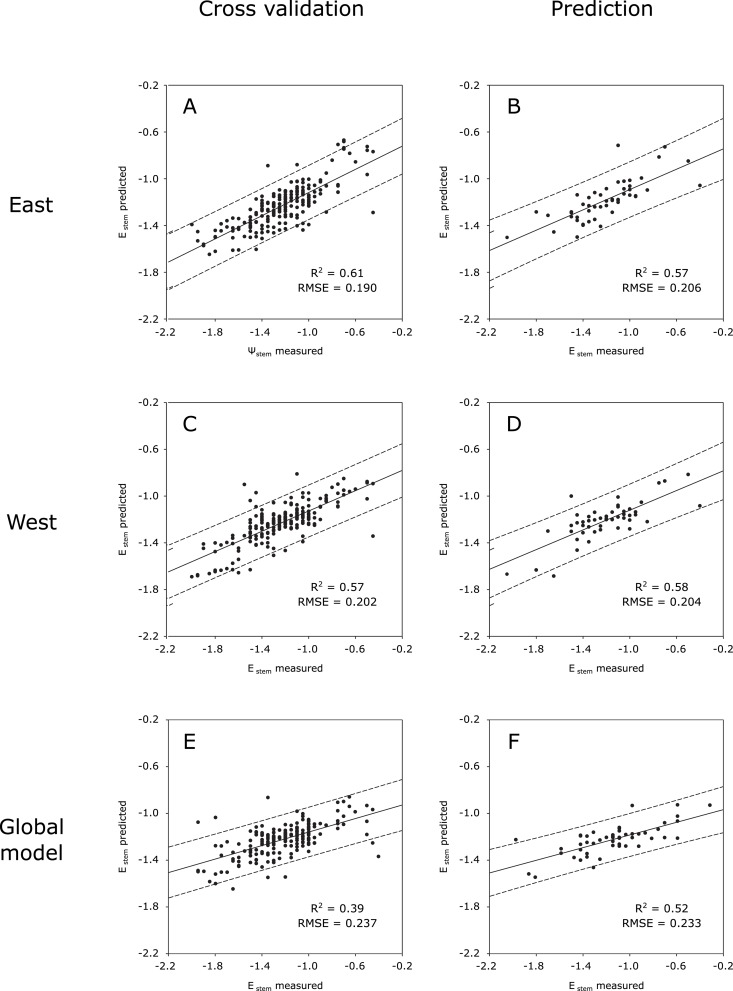
Regression plots for the water status prediction models with T_dry_ and T_wet_ reference temperatures. Plots from east (A, B) and west (C, D) sides of the canopy and from the global model trained with data from both sides (E, F); and for cross validation (A, C, E) and prediction (B, D, F). The dashed lines are the prediction confidence bands at 95%. The dotted lines represent the 1:1 line with slope 1.

The regression plots for the cross validation and prediction results for east and west models are displayed in Fig 4. In Fig 4A, a high distribution along the measured Ψ_stem_ values was achieved, and few samples fell outside the 95% prediction bands. The regression plot of the cross validation from the west side (Fig 4C) followed the same trend as well, having a good distribution throughout the whole water potential range. Additionally, the higher spreading level of the samples from the 1:1 line in Fig 4C drove the increased RMSE value of the west side model presented in Table 2.

The prediction plots for both east and west sides (Fig 4) displayed a virtually identical behavior, in terms of determination coefficient and root mean square errors. In Fig 4B, all the samples but one were located within the 95% prediction bands, and a wide coverage throughout the Ψ_stem_ range was scored (values from -2.1 to -0.4 MPa). Regarding the prediction plot considering the west side of the canopy, two samples lied outside the prediction bands, but a very similar fit around the regression line was achieved by the samples, casting a coincident R^2^ value (Table 2).

A statistical t-test analysis of the slopes showed no significant differences between the equations of each side for both cross validation (*p* = 0.47) and prediction (*p* = 0.86).

### Vineyard water status prediction models without T_dry_ and T_wet_ reference temperatures

Table 3 shows the statistics obtained by the stem water potential models that were trained without the reference temperatures.

**Table 3:** Performance statistics for the stem water potential (Ψ_stem_) prediction models without the use of T_dry_ and T_wet_ reference temperatures.

	N = 200				N = 50	
	Calibration		Cross validation		Prediction	
Canopy side	R^2^	RMSE	R^2^	RMSE	R^2^	RMSE
**East**	0.83	0.136	0.59	0.195	0.60	0.191
**West**	0.81	0.142	0.62	0.190	0.65	0.184
**Global**	0.75	0.170	0.45	0.224	0.56	0.216

A 10-fold cross validation was used.

N: number of samples. RMSE: root mean square error (MPa). East: dataset having all the samples from the east side of the canopy. West: dataset having all the samples from the west side of the canopy. Global: dataset having non-repeated samples from both east and west sides of the canopy.

The determination coefficients R^2^ obtained for the calibration process were above the 0.80 mark (0.83 for east and 0.81 for west), with RMSEs of 0.136 and 0.142 MPa, respectively. R^2^ values around 0.60 were achieved in the cross validation, with very similar RMSE values around 0.190 MPa for both canopy sides. The prediction results were slightly better than those of cross validation, in terms of R^2^ and RMSE: 0.60, 0.191 MPa (east side) and 0.65, 0.184 MPa (west side, Table 3). The performance statistics obtained from the global models were lower than those from the site-specific datasets.

Comparing these outcomes with those from the models trained with the reference temperatures, the statistics were very similar in the calibration and cross validation, but the models without T_dry_ and T_wet_ brought better R^2^ and RMSE values in the prediction. In the calibration, both kind of models achieved the same R^2^ values (and almost identical RMSEs), while in the cross validation the best statistics came from the west side models, being the opposite in the models with reference temperatures (Table 2). Still, these values followed the same overall trend.

**Fig 5:**
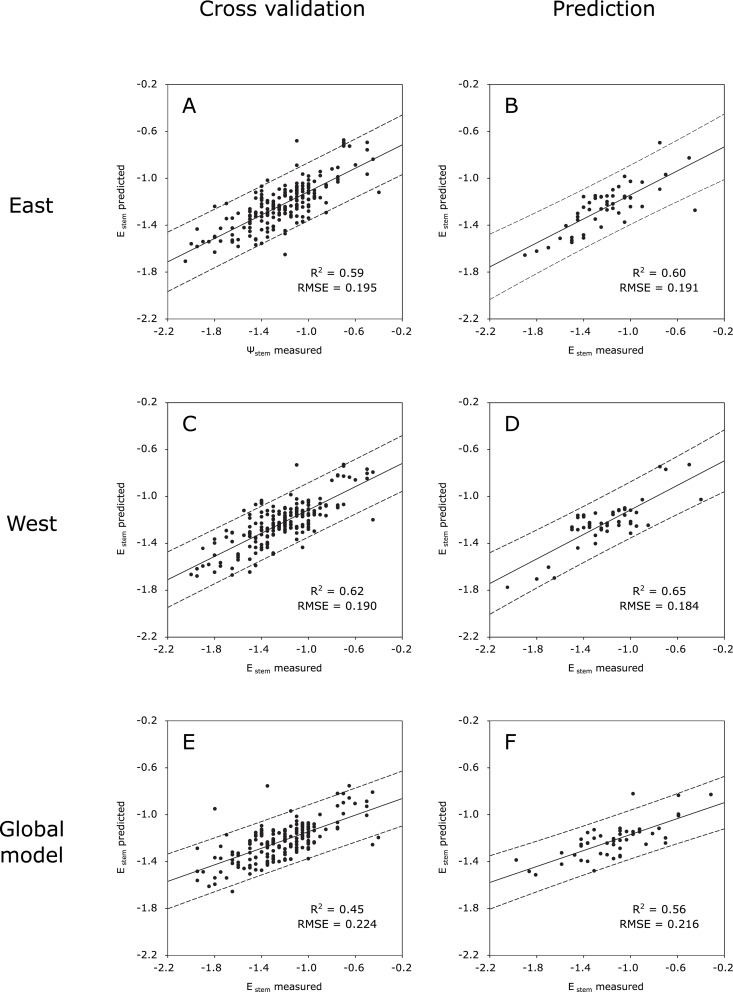
Regression plots for the water status prediction models without thermal indices. Plots from east (A, B) and west (C, D) sides of the canopy and from the global model trained with data from both sides (E, F); and for cross validation (A, C, E) and prediction (B, D, F). The dashed lines are the prediction confidence bands at 95%. The dotted lines represent the 1:1 line with slope 1.

The regression plots for the cross validation and prediction models trained without T_dry_ and T_wet_ reference temperatures (for east, west and both sides) are grouped in Fig 5.

The cross validation outcomes covered a high Ψ_stem_ range, with samples along the whole range (Fig 5A and 5C) and similar distribution around the 1:1 line. This resulted in similar RMSE values (Table 3). Additionally, the R^2^ values were nor far from each other, driven by the almost identical trend from the regression lines (as no significant differences were found between the slopes of both sides’ equations, for both cross validation—*p* = 0.93—and prediction—*p* = 0.89).

In Fig 5B and 5D, displaying the regression plots for the prediction results, higher determination coefficients can be found comparing to those from the prediction outcomes of the models trained with the reference temperatures (Fig 4B and 4D). Nevertheless, contrary to the latter, in this case the best results came from the west side of the canopy instead of the east side, with a higher fit to the regression line (meaning a higher R^2^ value) and a lower RMSE value (meaning a lower average distance of the samples from the 1:1 line).

## Discussion

The results presented in this study highlight the actual prospect of obtaining on-the-go, fast assessment of a vineyard’s water status directly on the field using proximal thermal imaging and machine learning algorithms. On-the-go assessment has a big advantages *vs*. other developed techniques for plant water status assessment or direct measurement of plant-based reference parameters [4], that are slower, manually controlled and with a lower capability of automatically characterizing a whole vineyard plot. It has also been proven that machine learning models, trained with data from seven days along a whole campaign, were capable of yielding plant water status prediction without the need of taking previous reference temperatures. This would allow a fastest, direct usage of this solution for in-field scenarios. The outcomes obtained using the models trained with reference temperatures demonstrated that canopy temperatures, along with T_dry_ and T_wet_ and weather parameters (such as the air temperature), would be very suitable for the useful prediction of the vineyard water status. Furthermore, the fact that similar results in cross validation (and even better outcomes in prediction) were achieved by the models trained without reference temperatures, brought high robustness into the models. This could allow to affirm that their implementation in industry and commercial environments is greatly feasible, also removing the necessity of the reference temperature measurement phase—and the calculation of thermal indices.

The relation between environmental status and canopy thermal response was clearly displayed throughout the whole experiment. Incoming solar irradiance casted high values most of the days, positively correlating with the high temperatures of the canopy in both sides. Nevertheless, two specific dates did not follow this trend, as in July 20th the canopy temperatures were particularly high for the relatively low irradiance received, and in August 11th the temperatures stayed very low regardless of the high amount of solar irradiance captured. This behavior seemed to be in contradiction with the expected response, but it is clearly explained by the VPD factor. As previously observed by other authors [11, 22, 41–43], VPD has a strong relationship with stomatal conductance and canopy temperature. In the case of July 20th, in spite of the low irradiance value, the increase of leaf temperature was mainly driven by the increase of VPD. Likewise, when the VPD, hence the evaporative demand increases, so does the actual rate of water vapor exchange through the leaf stomata. In this situation, the plant regulates this water loss by closing their stomata, leading to leaf temperature increase. On the other hand, in August 11th the low VPD value may not have led to stomata closure, hence leaf evapotranspiration was not impaired, and let the plants to cool down their leaf surface. Reported in other works [11, 41, 44], it exists a general correlation between VPD and water status, as higher VPD values limit the plants’ capability of a proper transpiration, although in the case of the present study the plant water status is mainly steered by the applied water regimes rather than the VPD. All these physiological behaviors confirm the usefulness of measuring the canopy temperature to assess the water status of a vineyard.

A good effectiveness was accomplished in canopy side-specific models. The vineyard plot in which the experiment was conducted had a North-South row orientation, so—at measurement time (between 14:00 and 15:00, local time)—the east side was shaded but with a whole morning of direct sunlight accumulated, while the west side started an afternoon of illumination from the sun. The row orientation does have a direct impact upon the canopy temperature and, thus, upon the plant response and acclimation [45]. Therefore, the development of two distinct models for each side of the canopy was a necessary step to allow the machine learning algorithms to adapt their rule extraction ability to the specific canopy thermal information that was present in each face. The fact that the global model provided poorer results could be explained by an assumed confused information given to the algorithms, as the plant thermal response would be very different for each side of the canopy. Still, the side-specific models were able to successfully develop adequate prediction rules for the two datasets and to cast similar performance statistics, demonstrating the possibility of a fairly acceptable water status estimation of a vineyard if the thermal measurements are taken from the same side of the canopy. In a real scenario, the development and implementation of two canopy side-specific models could be totally feasible and their application would only depend on the side of the input data.

The use of machine learning approaches for the grapevine water status estimation proved to be effective. It is visible the high suitability of machine learning algorithms for the solution of specific problems—grapevine water status prediction—but from very different kinds of input—spectral information and thermal imaging. In the case of the latter, the variables used (thermal and weather data) were selected in order to provide a decent amount of information from a thermal image. Although small differences would be present between two of the attributes used, mean and median of the canopy temperature, they could be sufficient for the algorithms to extract informative rules from them. Moreover, the use of principal component analysis by rotation forests would deal with potential cases of collinearity. Additionally, a desirable behavior was obtained from the machine learning models. The fact that considerably similar results were casted by cross validation and prediction increases the reliability of the techniques in their appraisal ability.

The relationship between leaf temperature—taken directly in the field—and water status of different kind of crops has been widely studied in diverse research works. For example, airborne thermal imaging has been used for the mapping of cotton water status (represented by the leaf water potential Ψ_leaf_) [17], and the same authors attempted the estimation of water status of the same kind of crop using ground-based thermal images [16], obtaining good results. Although the models presented in the present study returned lower performance statistics than those from [16, 17], the prediction capability of the machine learning algorithms was greatly supported by the high amount of data and its wide variability. The input samples employed in the training of the models (from very different phenological stages) were able to develop a whole prediction model capable of making Ψ_stem_ predictions in different moments within a wide time period. In grapevine, as in the present work, CWSI along with air temperature were used for the mapping of the water status (Ψ_leaf_) of a Pinot noir vineyard [21]. The same authors also studied the effect of different phenological stages in the evolution of the CWSI in grapevines [22]. In [46], the vineyard water status variability was monitored using aerial thermal and multispectral imagery. These studies, providing R^2^ values from 0.50 to 0.73, along with the results presented in the current research work, confirm the strong correlation of the canopy temperature with the grapevine water status, being possible to find underlying rules for the appraisal of an important reference value, such as water potential. The results reported by other authors from aerial thermal imaging of grapevines, as in [23, 24] or [46] also opened the possibility of estimate water status using thermal information from a lateral view, that would allow to gather a richer amount of information than that from zenith point of view. Some comparable works based on lateral thermal imaging but in static and manual approaches can be found in the literature. In [19] and [20], the authors used thermal imaging laterally acquired in a commercial vineyard to correlate the thermal indices CWSI and I_g_ with different plant water status reference parameters, with R^2^ values up to 0.78 and 0.70. The results from the on-the-go estimation of the grapevine water status represent an improvement not only regarding the possibility of quickly assessing and mapping a whole vineyard plot (instead of taking isolated thermal measurements), but also presenting a robust model trained with a collection of a high number of samples from different dates, and additionally removing the necessity of acquiring reference temperatures, using only the data provided by the canopy and air temperature.

The main pitfalls of other developed techniques plant water status assessment, such as the necessity of supervised measurement or the hard adaptation for the characterization of a whole vineyard plot makes on-the-go approaches a very suitable candidate for grapevine monitoring. The on-the-go proposal detailed in this work opens a large number of new opportunities in the implementation of this technological solution for the estimation of grapevine water status. Contrary to the developments that can be found in other related works, as in [19, 47, 48] or [49], involving manual and static measurements, the acquisition of thermal images from a moving vehicle expands—in the context of precision viticulture—the possibility of monitoring a whole vineyard plot in a faster and automated way. The delineation, for example, of different zones with homogeneous irrigation regimes could be feasible, and it could save higher amounts of water and money. Other plant water status estimation studies carried out with thermal imaging made use of information from aerial sources ([16, 17, 20–24]). Still, the acquisition of canopy side-aimed data that covers a larger area may assure a higher robustness in the given prediction. Additionally, the temporal flexibility of the approach from the present work arises as an advantage *vs*. airborne solutions, due to the fact that aerial acquisitions are very subjected to meteorological conditions and legal requirements. An on-the-go implementation of the proposal in the present work would be easily deployed directly in the field. A thermal system could be installed in an agricultural vehicle (e.g. a tractor conducting different viticulture operations), making constant measurements and giving real-time information to the driver or the manager. This possibility of covering large areas of a vineyard also makes available, jointly to an attached global positioning system, the creation of thermal-based maps of the plot that could provide detailed information about the water status of the monitored zones, making it an useful tool for irrigation scheduling in the decision-making process.

## Conclusion

The present work introduced a new methodology for the on-the-go assessment of vineyard water status using thermography and machine learning algorithms. The results obtained open a way for the implementation of a vineyard water status appraisal system available to be set up in a moving vehicle, given the fact that the use of data from a whole campaign for the training of the models brought stronger reliability. Also, good prediction results has been achieved without the need of reference temperatures, thus removing the requirement of the supervised acquisition of these values. This advantage could clear new paths in sustainable viticulture, making possible the deployment of solutions that could characterize in an automatic and continuous way a whole vineyard for a more accurate application of irrigation in viticulture, that is a very needed requirement in the current context of climate change and water scarcity.
